# Pisa syndrome induced by switching of a choline-esterase inhibitor treatment from donepezil to galantamine: a case report

**DOI:** 10.1186/s12883-020-01769-2

**Published:** 2020-05-13

**Authors:** Yu Mimura, Shin Kurose, Taketo Takata, Hajime Tabuchi, Masaru Mimura, Michitaka Funayama

**Affiliations:** 1 Department of Neuropsychiatry, Japanese Red Cross Ashikaga Hospital, 49-1 Yobe, Ashikaga, Tochigi, Japan; 2 Department of Neuropsychiatry, Keio University School of Medicine, 35 Shinanomachi, Shinjuku, Tokyo, Japan

**Keywords:** Pisa syndrome, Choline-esterase inhibitor, Galantamine, Dopamine, Nicotinic effects, Extra-pyramidal symptom, Alzheimer’s disease

## Abstract

**Background:**

Pisa syndrome (PS) is characterized by an abnormally sustained posture, with flexion of the body and head to one side and slight rotation of the trunk. Although PS most commonly arises as an adverse effect of antipsychotic drugs, choline-esterase inhibitors (ChEIs) are also sometimes known to induce PS. Despite the fact that the precise mechanism remains unclear, cholinergic-dopaminergic imbalance has been considered as a possible pathophysiologic mechanism underlying the genesis of PS.

**Case presentation:**

We hereby report the case of a 60-year-old woman with Alzheimer’s disease who presented with the signs of PS after her treatment was switched to galantamine, a type of ChEI, even though she had received donepezil, another type of ChEI, for 5 years without any complications. To the best of our knowledge, this is the first report of PS associated with treatment switch from one to another type of ChEI. Galantamine, but not other ChEIs, can enhance striatal dopamine release through allosteric modulation of the nicotinic acetylcholine receptor, and has weaker muscarinic effects than donepezil. Therefore, we propose two novel hypotheses to explain the development of PS, as follows; galantamine, which enhances dopamine release, can induce imbalance of dopamine levels in the striatum of patients with dementia, resulting in PS, and the weaker muscarinic effects of the drug could be one of the factors predisposing to the development of PS.

**Conclusion:**

The present case suggests that treatment with galantamine is associated with a higher risk of development of PS than that with other ChEIs, such as donepezil, despite the pharmacological profile of galantamine as a dopamine modulator. Also, this report provides novel insight into another plausible mechanism underlying the development of PS, besides cholinergic-dopaminergic imbalance, namely, dopamine imbalance in the striatum with muscarinic-nicotinic imbalance.

## Background

Pisa syndrome (PS) or pleurothotonus, is characterized by a marked lateral trunk flexion that can be reduced by passive mobilization or supine positioning [1], and was originally described by Ekbom in 1972 [2]. PS is observed in patients with neurodegenerative diseases, mainly Parkinson’s disease [3]. PS is also considered as one of the rare types of tardive dystonias caused by drugs such as choline-esterase inhibitors (ChEIs) [4–18], antipsychotics [19–21], antidepressants [22, 23], lithium [24], and valproic acid [25]. However, the precise pathophysiology of PS has not yet been established. PS induced by ChEIs and antipsychotics has been assumed to be induced by cholinergic-dopaminergic imbalance. In other words, antipsychotics can decrease dopaminergic neurotransmission, and ChEIs can increase both the levels and actions of acetylcholine in the synaptic clefts to cause choline-dominance imbalance [3, 11, 17]. Decrease of dopaminergic functions with enhanced cholinergic functions cause the tonic influence on posture and locomotion to change toward the direction of immobility, because cholinergic-dopaminergic balance in the nigrostriatal neuronal system maintains normal muscle tone in the human body [26]. Disruption of the cholinergic-dopaminergic balance could result in an asymmetric axial muscle tone activation, and this is the hypothesized pathogenic mechanism underlying the development of drug-induced PS. Herein, we present the case report of a patient who presented with signs of PS following switching of ChEI treatment from donepezil to galantamine. Our case might shed some light on the onset of PS induced by ChEIs.

## Case presentation

A 57-year-old Japanese woman visited the memory clinic affiliated to our hospital with a 2-year history of visual memory loss. Examination revealed that the patient had agraphia as well as left-right agnosia. Her insights into her cognitive dysfunction, however, were relatively well-preserved. She showed no signs of parkinsonism. Magnetic resonance imaging and single- photon emission computed tomography of the head revealed bilaterally symmetric atrophy of the occipitoparietal lobes and decreased blood flow to the same areas. She was clinically diagnosed as having posterior cortical atrophy, a visual variant of early-onset Alzheimer’s disease.

She was prescribed donepezil at the dose of 3 mg per day, which was later increased to 10 mg per day, in the absence of any side effects. Her visuospatial function gradually deteriorated during the treatment. In the following year, she presented with dressing apraxia. At the age of 60, she had difficulty in positioning herself to sit on a chair; her attempts to take a seat often resulted in her missing the chair and she found herself trying to sit on air instead. She was unable to find her way out of our examination room. She became dependent for her activities of daily living. She also became so impulsive and agitated that she was always talking to herself, without daily fluctuations in cognitive functions. Donepezil was discontinued in view of her agitation. Instead, she was started on augmentation therapy with the combination of galantamine and memantine to improve her psychiatric symptoms and maintain her cognitive function status. However, two weeks after the treatment switch from donepezil to galantamine plus memantine, she was admitted to the neuropsychiatric unit of our hospital, because her psychiatric symptoms did not improve, and she began to show signs of parkinsonism.

On admission, she presented with frozen gait and a mask-like expression. No rigidity, tremor or other neurological signs, such as paralysis or sensory impairment, were apparent. There were no remarkable changes of the vital signs or abnormalities on physical and laboratory examinations. Cerebrospinal fluid (CSF) examination revealed no increase of the cell count or protein levels, and the IgG index was within normal limits. The CSF amyloid beta 1–42(Aβ 1–42) level was 116 pg/mL and that of phosphorylated tau(P-tau) was 41 pg/mL, consistent with the diagnosis of Alzheimer’s disease [27]. To examine the possibility of Lewy body disease, ^123^I-(3meta)-iodobenzylguanidine myocardial scintigraphy and dopamine-transporter scanning were performed, which showed early and delayed heart/mediastinum ratios of 3.96 and 3.88, and specific binding ratios of 5.76 (right) and 5.78 (left), respectively, excluding Lewy body disease. Also, corticobasal syndrome was ruled out, because of the absence of laterality.

**Fig. 1:**
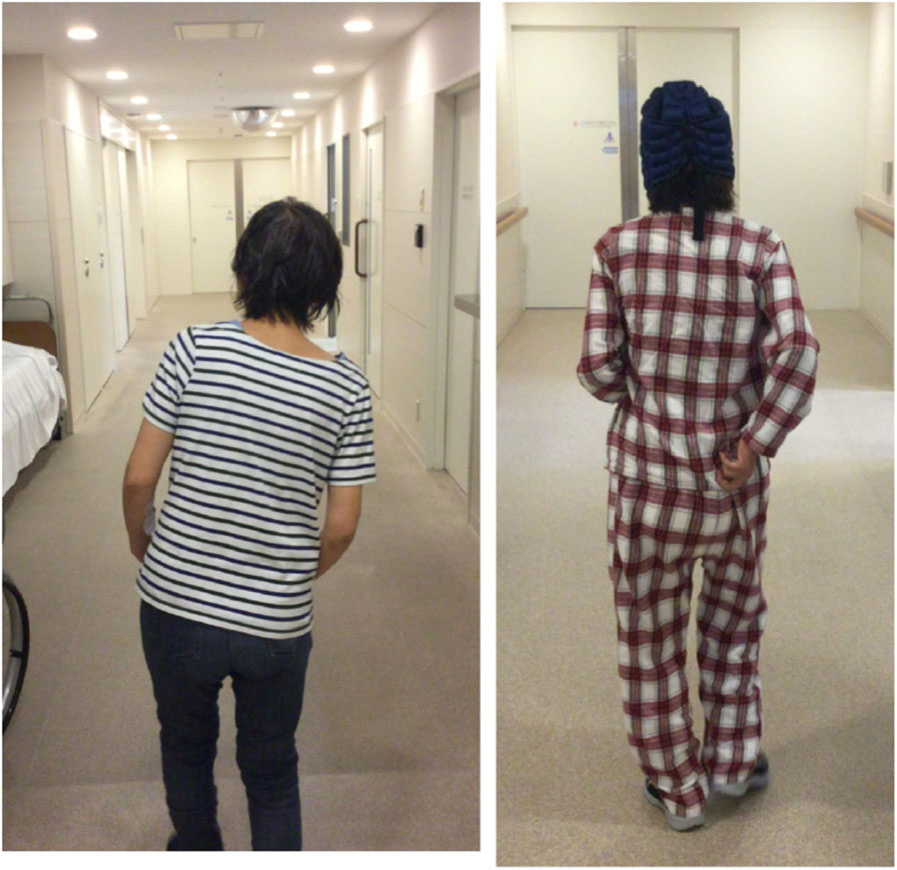
Left: Sustained flexion of both head and body trunk, with slight rotation of the body to right side. Right: 14 days after discontinuation of galantamine while continuing memantine therapy, the characteristic posture of Pisa syndrome disappeared

Her medications on admission included galantamine 4 mg bid, memantine 10 mg qd, suvorexant 20 mg qd. Her parkinsonism signs deteriorated after she was hospitalized. In particular, she showed sustained flexion of both the head and body trunk, with slight rotation of the body to the right side, consistent with the diagnosis of PS, which was exacerbated while walking; these symptoms remained fixed until the 13th hospital day. Based on the suspicion that the PS in this patient had developed as a side effect of galantamine, this drug was stopped on the 13th hospital day. After discontinuation of galantamine while continuing memantine monotherapy, the characteristic posture of PS gradually improved within 2–3 days. In accordance with improvements on posturing, her freezing of gait also disappeared so that she was able to walk without any assistance. Meanwhile, her masked face was preserved. Finally, her posturing almost thoroughly disappeared over the subsequent 14-day period (Fig. 1).

For the agitation and insomnia, the patient was prescribed 5 mg of asenapine, an antipsychotic, which was effective without any side effects in terms of extrapyramidal symptoms (EPS), including PS. By the 93rd hospital day, her neuropsychiatric symptoms had markedly improved and the patient was discharged home, where she was taken care of by her family members in her daily life.

## Discussion and conclusion

To the best of our knowledge, this is the first case report of PS induced by a treatment switch of a ChEI to galantamine, despite prior long-term use, for 5 years, of donepezil without any complications. As for PS induced by ChEIs, a large pharmacovigilance study reported 52 cases of PS [28]. According to the previous report, PS occurs most frequently with galantamine, followed by rivastigmine and donepezil [28]. Our case report on PS is consistent with their findings. We reviewed and summarized all case reports that described PS linked to ChEIs initiation in Table 1.

**Table 1:**
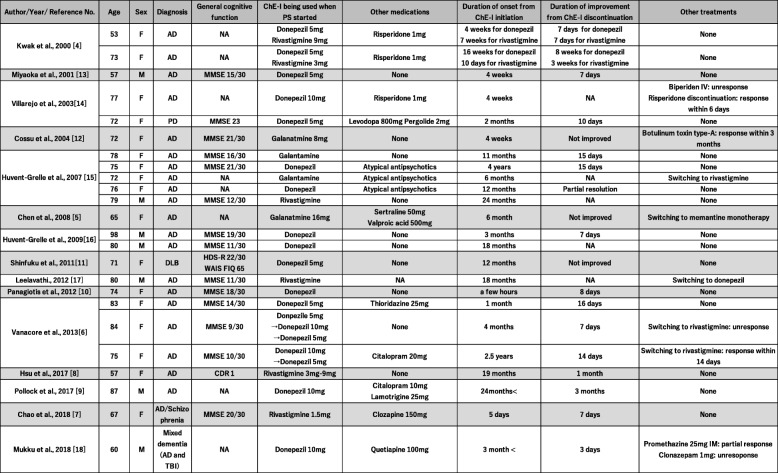
All previous case reports that described PS linked to ChEIs initiation in Table 1

The mechanisms underlying the development of PS are not yet fully understood. Many past reports have suggested that cholinergic-dopaminergic balance in the direction of cholinergic dominance is the main cause of PS [3]. This hypothesis is widely accepted and based on the fact that the symptoms most often develop with the use of antipsychotics and ChEIs, and improve with the use of anticholinergics [3].

However, the notion that galantamine, among the ChEIs is associated with the highest risk of development of PS is inconsistent with the results of a recent animal study on the effects of galantamine. Galantamine is not only a ChEI, but also an allosteric potentiating ligand (APL) for the nicotinic acetylcholine receptor. In experiments conducted using a hemiparkinsonian rat model, galantamine was found to facilitate striatal dopamine release by acting as an APL at the α4 nicotinic acetylcholine receptor [29]. This seems to be inconsistent with galantamine as a risk factor for PS, because EPS are primarily caused by blockade of the dopamine D2 receptor. On the other hand, in the experiment conducted by Inden et al., both methamphetamine, which enhances dopamine release, and the combination of a dopamine reuptake inhibitor plus galantamine induced the same rotational behavior, indicating that galantamine enhances dopamine release on the contralateral side of the striatum in the hemiparkinsonian model of rats [29]. From this point of view, dopamine imbalance triggered by galantamine in the striatum would induce rotation and flexion of the body to one side like PS. In our presented case, even though the dopamine transporter scan was negative, this imbalance might become worse by dopamine release induced on one side by galantamine.

In another mouse study conducted by Shimizu et al., the results were consistent with galantamine as a strong risk factor for PS [30]. According to this study on the effects of donepezil-, galantamine-, and antipsychotic drug-induced EPS, galantamine induced and augmented EPS more potently than donepezil [30]. In this experiment, EPS induced by galantamine was completely ameliorated by a muscarinic antagonist, but not by a nicotinic antagonist, suggesting that ChEI-induced EPS primary involves muscarinic effects and is improved by muscarinic antagonists. In addition, galantamine has a weaker antagonistic effect against the muscarinic receptor as compared to donepezil [31]. Taken together, weaker effect on the muscarinic receptors is associated with an increase in the risk of development of EPS.

In our case, memantine was the better alternative, because the patient showed good tolerability to memantine. In order to not only maintain the patient’s cognitive functions, but also to resolve the symptoms, memantine, which acts by non-competitive binding to the N-methyl-D-aspartate (NMDA) receptor, is an effective, reasonable, and safe treatment agent [5]. In fact, mouse experiments support this notion, in that memantine significantly reduced EPS induced by haloperidol, as compared to galantamine and donepezil, through its antagonistic effect on the NMDA receptor [32].

In addition to switching ChEIs, the patient’s impaired visuo-spatial function might be related to PS in the presented case. According to previous reports, visuo-spatial dysfunction assessed by Benton’s judgement line orientation test was a strong and significant predictor for developing PS [33, 34]. Given the abnormal posture in PS, however, disruption in body schema, rather than visuo-spatial dysfunction, might influence the development of PS because the main atrophic area involved the bilateral parietal lobe, which is considered the neural basis for body schema [35]. Still, switching was a major cause because posturing was completely resolved after discontinuation of galantamine.

This case report has some limitations. First of all, no electromyography was performed, because we were not sure whether we could perform the examinations safely. Also, there is a possibility that the symptoms represented delayed effects of donepezil in our case. However, we are reasonably certain about our diagnosis based on the fact that our patient presented with the symptoms of PS soon after (2 weeks later) her treatment was switched to galantamine and her symptoms disappeared soon after (2 weeks later) galantamine was discontinued. The Naranjo score of 5 indicates that galantamine was the probable cause of PS in our case [36]. Additionally, to the best of our knowledge, the longest period between the initiation of ChEI treatment and the onset of PS is 24 months [14]. Finally, a major limitation is that this was just a single case report. Further cases should be collected to better understand the pathophysiology of PS.

In conclusion, we present the first case report of PS that developed soon after the patient’s treatment was switched to galantamine from donepezil, even though the patient had shown good tolerability to 10 mg of donepezil for 5 years. This case suggests additional possible mechanisms underlying the development of PS, besides the conventional cholinergic-dopaminergic imbalance theory, including dopamine imbalance in the striatal system and muscarine-nicotine imbalance.

## Data Availability

All data generated or analysed during this study are included in this published article and its supplementary information files.

## Abbreviations

PS: Pisa syndrome
ChEIs: Choline-esterase inhibitors
CSF: Cerebrospinal fluid
APL: Allosteric potentiating ligand
EPS: Extrapyramidal symptoms
NMDA: N-Methyl-D-Aspartate
M: Male
F: Female
AD: Alzheimer’s disease
PD: Parkinson’s disease
DLB: Dementia with Lewy body
TBI: Traumatic brain injury
NA: Not available
HDS-R: Hasegawa Dementia Scale Revised
WAIS: Wechsler adult intelligence scale
FIQ: Full scale intelligence quotient
CDR: Clinical dementia rating
